# Nonlinear and delayed impacts of climate on dengue risk in Barbados: A modelling study

**DOI:** 10.1371/journal.pmed.1002613

**Published:** 2018-07-17

**Authors:** Rachel Lowe, Antonio Gasparrini, Cédric J. Van Meerbeeck, Catherine A. Lippi, Roché Mahon, Adrian R. Trotman, Leslie Rollock, Avery Q. J. Hinds, Sadie J. Ryan, Anna M. Stewart-Ibarra

**Affiliations:** 1 Department of Infectious Disease Epidemiology, London School of Hygiene & Tropical Medicine, London, United Kingdom; 2 Centre for the Mathematical Modelling of Infectious Diseases, London School of Hygiene & Tropical Medicine, London, United Kingdom; 3 Barcelona Institute for Global Health (ISGLOBAL), Barcelona, Spain; 4 Department of Public Health, Environments and Society, London School of Hygiene & Tropical Medicine, London, United Kingdom; 5 Centre for Statistical Methodology, London School of Hygiene & Tropical Medicine, London, United Kingdom; 6 Caribbean Institute for Meteorology and Hydrology, St. James, Barbados; 7 Quantitative Disease Ecology and Conservation Lab Group, Department of Geography and Emerging Pathogens Institute, University of Florida, Gainesville, Florida, United States of America; 8 Ministry of Health, St. Michael, Barbados; 9 Caribbean Public Health Agency, Port of Spain, Trinidad and Tobago; 10 School of Life Sciences, University of KwaZulu-Natal, Durban, South Africa; 11 Institute for Global Health and Translational Science, SUNY Upstate Medical University, Syracuse, New York, United States of America; 12 Department of Medicine and Department of Public Health and Preventative Medicine, SUNY Upstate Medical University, Syracuse, New York, United States of America; Africa Program, UNITED STATES

## Abstract

**Background:**

Over the last 5 years (2013–2017), the Caribbean region has faced an unprecedented crisis of co-occurring epidemics of febrile illness due to arboviruses transmitted by the *Aedes sp*. mosquito (dengue, chikungunya, and Zika). Since 2013, the Caribbean island of Barbados has experienced 3 dengue outbreaks, 1 chikungunya outbreak, and 1 Zika fever outbreak. Prior studies have demonstrated that climate variability influences arbovirus transmission and vector population dynamics in the region, indicating the potential to develop public health interventions using climate information. The aim of this study is to quantify the nonlinear and delayed effects of climate indicators, such as drought and extreme rainfall, on dengue risk in Barbados from 1999 to 2016.

**Methods and findings:**

Distributed lag nonlinear models (DLNMs) coupled with a hierarchal mixed-model framework were used to understand the exposure–lag–response association between dengue relative risk and key climate indicators, including the standardised precipitation index (SPI) and minimum temperature (Tmin). The model parameters were estimated in a Bayesian framework to produce probabilistic predictions of exceeding an island-specific outbreak threshold. The ability of the model to successfully detect outbreaks was assessed and compared to a baseline model, representative of standard dengue surveillance practice. Drought conditions were found to positively influence dengue relative risk at long lead times of up to 5 months, while excess rainfall increased the risk at shorter lead times between 1 and 2 months. The SPI averaged over a 6-month period (SPI-6), designed to monitor drought and extreme rainfall, better explained variations in dengue risk than monthly precipitation data measured in millimetres. Tmin was found to be a better predictor than mean and maximum temperature. Furthermore, including bidimensional exposure–lag–response functions of these indicators—rather than linear effects for individual lags—more appropriately described the climate–disease associations than traditional modelling approaches. In prediction mode, the model was successfully able to distinguish outbreaks from nonoutbreaks for most years, with an overall proportion of correct predictions (hits and correct rejections) of 86% (81%:91%) compared with 64% (58%:71%) for the baseline model. The ability of the model to predict dengue outbreaks in recent years was complicated by the lack of data on the emergence of new arboviruses, including chikungunya and Zika.

**Conclusion:**

We present a modelling approach to infer the risk of dengue outbreaks given the cumulative effect of climate variations in the months leading up to an outbreak. By combining the dengue prediction model with climate indicators, which are routinely monitored and forecasted by the Regional Climate Centre (RCC) at the Caribbean Institute for Meteorology and Hydrology (CIMH), probabilistic dengue outlooks could be included in the Caribbean Health-Climatic Bulletin, issued on a quarterly basis to provide climate-smart decision-making guidance for Caribbean health practitioners. This flexible modelling approach could be extended to model the risk of dengue and other arboviruses in the Caribbean region.

## Abstract 2

Rachel Lowe and colleagues model for the delayed effects of climate change on outbreaks of Dengue in Barbados, an approach that can be extended to model risk for other arboviruses in the Caribbean

## Author summary

**Why was this study done?:**

- Changes in local climate conditions (i.e., rainfall, temperature) can affect the risk of outbreaks of diseases transmitted by mosquitoes, such as dengue fever, chikungunya, and Zika.
- Climate information can be used to develop forecasts of disease outbreaks.

**What did the researchers do and find?:**

- In this study, we devised a statistical model to test whether dengue outbreaks in the Caribbean island of Barbados could be predicted using weather station data for temperature and a precipitation index—used to monitor drought and extreme rainfall—as model inputs from June 1999 to May 2016.
- The model was able to successfully predict months with dengue outbreaks versus nonoutbreaks in most years.
- The risk of dengue outbreaks increased with increasing minimum temperature (Tmin; up to 25°C). Disease outbreaks were more likely to occur 4 to 5 months after periods of drought and 1 month after periods of excess rainfall.
- The modelling results suggest that a drought period followed by intense rainfall 4 to 5 months later could provide optimum conditions for an imminent dengue outbreak.

**What do these findings mean?:**

- In practice, the Regional Climate Centre (RCC) could use this dengue model to generate disease forecasts using their seasonal climate forecast products.
- Public health decision-makers can use the dengue forecasts as an early warning tool to plan interventions to reduce the risk of dengue and other mosquito-borne diseases.
- The ability of the model to predict dengue outbreaks is complicated by the emergence of new diseases with similar symptoms that are transmitted by the same mosquito vector, including chikungunya and Zika.

## Introduction

Small Island Developing States (SIDS) in the Caribbean are among the most vulnerable countries to extreme climate events (e.g., droughts and tropical storms), which are becoming more frequent and severe due to climate change [1]. Climate events have a major impact on human health in the Caribbean, including impacts on arboviral disease transmission, heat-induced morbidity, water-borne diseases, injuries, respiratory complications, and mental health [2]. The social and economic cost of these adverse health outcomes is a major burden on SIDS, and the burden is projected to increase with the changing climate [3].

In recent years, the Caribbean region has experienced an unprecedented crisis of co-occurring epidemics of febrile illness due to dengue, chikungunya, and Zika viruses. These diseases are transmitted principally by the female *Aedes aegypti*, which is a mostly domestic, urban mosquito that lays its eggs in water-bearing containers in and around the home. Over a 5-year period (2012 to 2016), Barbados reported a total of 7,298 dengue cases [4], with most cases reported in children up to 15 years of age [5]. Three outbreaks of dengue fever were reported during that period (2013, 2014, and 2016). Recent studies reported an upward trend in dengue transmission in Barbados from 2006 to 2015, with all 4 dengue serotypes (dengue virus [DENV]-1 through DENV-4) cocirculating in the population [5]. Illness due to dengue virus is estimated to cost $321.4 million USD per year in the Caribbean (excluding the costs of vector control and other prevention programmes) [6]. With the recent emergence of chikungunya and Zika in the Caribbean, the cost of *Aedes*-transmitted diseases has increased dramatically. In a region with low economic growth and high public debt [7,8], the burden of arboviruses is unsustainable, and new tools are urgently needed to support the public health sector.

Prior studies have shown that climate variability influences dengue transmission and *A*. *aegypti* population dynamics in the Caribbean [9–12]. Ambient temperatures impact disease transmission by affecting mosquito development rates, reproduction, survival, biting rates, and viral replication in the mosquito [13]. Warmer temperatures increase the risk of disease transmission up to an optimum temperature range of 26 to 29°C [13]. The effect of rainfall on dengue risk is more complex and depends on the local socioecological conditions that determine the type and abundance of larval habitat in the environment [14]. Rainfall can increase mosquito population densities by increasing the availability of larval habitat in abandoned rain-filled containers in the patio around the home. Drought conditions that result in household water scarcity can also potentially increase larval habitat by increasing the number of water storage containers around the home [15]. However, few studies have examined the effects of prolonged drought on dengue transmission.

Prior studies in Barbados documented the seasonal linkages between climate and dengue (from 1980 to 2000), finding that epidemics occurred in the latter part of the year [16]. The study also found that there was a trend in increasing temperatures and declining rainfall in recent decades. Other studies found that dengue was significantly associated with lagged climate variables, including rainfall at a 7-week lag and minimum temperature (Tmin) at a 12-week lag [10]. In a different study, investigators analysed a time series of dengue from 2004 to 2013 using generalised linear models, which were able to predict dengue cases with limited success using monthly rainfall, monthly number of rain days, and average temperature, after pre-adjusting for unmeasured cyclical and long-term trends [17]. Most recently, investigators analysed monthly dengue cases from 2006 to 2015 and found a positive correlation between rainfall and relative humidity of the same month. However, they did not assess lagged relationships [5].

The El Niño Southern Oscillation (ENSO) is a major driver of regional year-to-year climate variability in the Caribbean [18,19]. ENSO is associated with a gradient in sea surface temperature anomalies between the eastern Tropical Pacific Ocean and the Tropical North Atlantic Ocean and Caribbean Sea. During the early and mature stages of El Niño events, this gradient is often positive (i.e., the surface waters of the Tropical Pacific are anomalously warmer than the Tropical North Atlantic and Caribbean Sea). Most periods of regional drought, weaker hurricane season activity, and warmer temperatures are associated with the El Niño–induced positive gradient. By contrast, wet episodes and more active hurricane seasons coincide with La Niña events [19], i.e., when the ocean gradient tends to be negative. Recent examples of El Niño–associated regional drought occurred in 1997–1998, 2009–2010, and 2014–2016, whereas La Niña events in 2010–2011 and 2011–2012 coincided with very wet conditions in large parts of the Caribbean [20]. In Barbados, we hypothesise that the most important extreme climate events with respect to arbovirus risk are droughts, which extend into the dry season when water availability is reduced [20], and periods of excessive rainfall during the wet season.

Before an effective disease forecast can be developed, it is important to identify the key climate drivers, lag periods, and appropriate model formulation that reflects the local disease transmission ecology and climatology. It takes time for anomalies in the climate to manifest and contribute to disease risk. The various components of the time lag include the period for mosquito larval habitat to increase, the development period of the mosquito, the time before the first blood meal in which the mosquito transmits the virus to a human host, and the time before the appearance of clinical manifestations of dengue [10]. Several studies have employed distributed lag nonlinear models (DLNMs) to explore associations between meteorological variables and dengue incidence in several countries—primarily in Asia—that experience dengue epidemics, including Bangladesh [21], China [22], Indonesia [23], Malaysia [24], Singapore [25], Sri Lanka [26], and Taiwan [27]. A major advantage to using DLNM methodology is the possibility of describing the lag structure of either linear or nonlinear exposure–response relationships, through the choice of 2 functions that define the association along the dimensions of the predictor (e.g., climate variables) and lags [28]. The analysis of the temporal evolution of disease risk associated with protracted time-varying climatic exposures has implications for the development of climate-based disease early warning systems.

This study aims to quantify the nonlinear and delayed effects of climate impacts, such as drought, extreme rainfall, and temperature variations, on dengue risk in the eastern Caribbean island of Barbados by coupling DLNMs with a Bayesian model estimation framework. The model is then used to produce out-of-sample predicted probabilities of exceeding island-specific outbreak thresholds. Barbados is an ideal case study due to high-quality historical climate and health data, a relatively high burden of disease, and prior experience in building projects on climate and health, paving the way for a sustainable collaboration to develop integrated early warning information to predict the risk of dengue and other mosquito-transmitted diseases in the Caribbean.

## Materials and methods

### Study area

This study was carried out for the Caribbean island of Barbados (13° N 59° W) in the Lesser Antilles. Barbados is relatively small in land area (425 km^2^), with a resident population of over 277,000 people [29]. Due to a thriving tourism-based economy, over 1 million people visit Barbados each year [30]. The island is administratively divided into 11 parishes, the most densely populated being the urban parish of St. Michael, which contains the capital city of Bridgetown (see S1 Fig). Dengue is one of the primary febrile illnesses in Barbados, especially during the wet season, when the mosquito vector is most abundant. *A*. *aegypti* is the only known vector on the island.

### Health data

The surveillance unit of the Environmental Health Department in the Ministry of Health of Barbados is responsible for maintaining records on the incidence of dengue and other arboviral disease cases. As of January 2018, the surveillance unit liaises with the Leptospirosis lab (national reference lab) to record laboratory-confirmed cases of dengue virus, chikungunya virus, and Zika virus. Specimens are tested for the dengue virus nonstructural protein 1 (NS1) antigen, dengue virus IgM/IgG, and chikungunya virus IgM/IgG using commercial ELISA kits. Since September 2016, the lab has also conducted real-time PCR using the CDC Trioplex assay for dengue virus, chikungunya virus, and Zika virus, and they conduct serotyping of positive dengue virus samples. National population estimates, obtained from the Barbados Population and Housing Census (1990, 2000, and 2010) [29], were used to estimate yearly population during the study period by linear interpolation. Population data were used to calculate monthly incidence rates of laboratory-confirmed dengue cases in Barbados from June 1999 to May 2016.

### Climate of Barbados

The climatology of Barbados fits well within that of the Lesser Antilles. The wet season lasts from June until November and coincides with the Atlantic Hurricane Season [31]. The dry season occurs from December to May. More than 70% of annual rainfall totals are recorded on average during the wet season [32], which is further characterised by elevated relative humidity, increased rainfall frequency and intensity, and the irregular occurrence of spells of very heavy rainfall [31]. The wet season typically features 2 to 3°C higher dry-bulb temperatures than the relatively cool period of December to February, with heat waves becoming more frequent in recent decades [31,33].

Given its low topography, Barbados records significantly less rainfall, on average, than neighbouring mountainous islands, from less than 1,200-mm rainfall per annum in some low-lying areas to around 2,000-mm per annum above 300-m elevation [31]. S2 Fig shows the 1981–2010 average rainfall seasonality in terms of rainfall chance and rainfall intensity on wet days for the synoptic station located at the CIMH. The combination of relatively little rainfall, high population density, and a mostly urban population makes Barbados a particularly water-scarce country [34].

### Meteorological data

Monthly rainfall totals and average minimum, mean, and maximum temperatures were extracted from the 2 synoptic weather stations on the island—at the Grantley Adams International Airport (GAIA), at an elevation of 56 m in the southern parish of Christ Church, and at the Caribbean Institute for Meteorology and Hydrology (CIMH), at an elevation of 112 m in the western parish of St. James (see S1 Fig and S3 Fig). Monthly minimum (maximum) temperature is defined as the arithmetic average of the daily minimum (maximum) dry-bulb temperature, measured under standard Stevenson screen conditions.

### Standardised Precipitation Index

The Standardised Precipitation Index (SPI) is a drought index first developed by McKee et al. [35]. The SPI is a representation of rainfall totals in units of standard deviation following a gamma-fitted distribution. Positive values indicate greater than average rainfall totals or excess rainfall, while negative values indicate less than average rainfall or rainfall deficits—otherwise called meteorological drought. The SPI can best be interpreted as the severity of meteorological drought or excessive rainfall by the end of the period of interest and is based on monthly precipitation totals alone—the most commonly available climate variable from rainfall stations around the world. As such, wet or dry conditions can be monitored by the SPI at a variety of time horizons, from subseasonal to multi-annual time scales.

SPI at different time scales are representative of different types of drought. Generally, the longer the meteorological drought persists and the more negative the SPI value, the greater the societal impact. The 1-month SPI (SPI-1) allows for early detection of meteorological droughts. Operationally, with the aim to enable drought early warning systems, the Caribbean Drought and Precipitation Monitoring Network (CDPMN)—coordinated by the CIMH—tracks drought using SPIs at 1 month, 3 months (SPI-3), 6 months (SPI-6), 12 months (SPI-12), and 24 months (SPI-24) [31] to cover the range of drought durations experienced in its climate [20]. For example, rainfall deficits over 1 month may be enough to dry the topsoil, especially during the latter part of the dry season. However, in the Caribbean, agricultural drought (related to shortage of surface or soil moisture for optimal crop growth) manifests more significantly after 3 months [20]. Therefore, SPI-3 and SPI-6 can be used to track agricultural drought. Soil moisture and surface water reservoir depletion typically take at least 6 months to substantially impact water availability at the national level. Aquifer depletion and repletion typically occur at time scales of 9 to 12 months, affecting large groundwater reservoirs. Therefore, hydrological drought can be tracked with SPI-6 and SPI-12.

With drought being a slow-onset climate hazard, early warning (e.g., based on SPIs) with ample lead time is a realistic and effective option. This is especially the case when monitoring is combined with forecasting. Operationally, the Caribbean Climate Outlook Forum (CariCOF) provides drought forecasts using predictions of SPI-6 with a lead time of 3 months (i.e., 3 months in advance) and SPI-12 with lead times up to 6 months [20]. In contrast, while SPIs are used by the CDPMN to track periods of excess precipitation, flooding potential—the hydrometeorological hazard component of flood risk—is a compounded factor of both long-term rainfall accumulations (i.e., soil moisture saturation) and short-term wet spells. The original severity classes are shown in S1 Table. Impacts experienced (particularly in agriculture) during the Caribbean drought of 2009–2010 led to a redefinition of the categories (see S2 Table), which has been used for decision-making since 2011.

For this study, all SPI values were calculated based on the records of daily rainfall totals at the CIMH and the GAIA stations in Barbados, which start in January of 1981 and 1971, respectively. The historical climatological reference was calculated using the period 1981–2010 to conform with World Meteorological Organization regulations.

**Fig 1:**
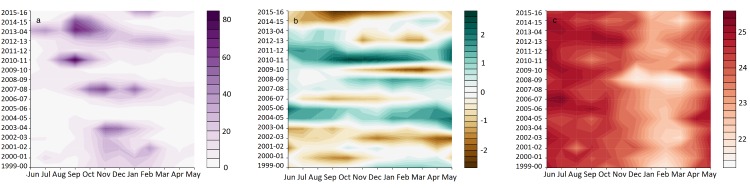
Annual cycle of dengue incidence rates, SPI-6, and Tmin June 1999–May 2016. Annual cycle of (a) dengue incidence rates (per 100,000 population) in Barbados, (b) SPI-6, and (c) Tmin (°C) averaged over CIMH and GAIA weather stations at the monthly time scale from June 1999 to May 2016. CIMH, Caribbean Institute for Meteorology and Hydrology; GAIA, Grantley Adams International Airport; SPI-6, 6-month Standardised Precipitation Index.

Fig 1 shows the annual cycle of dengue incidence rates (per 100,000 population), the SPI-6, and Tmin in Barbados from June 1999 to May 2016. Dengue outbreaks occurred in the dengue seasons 2003–2004, 2007–2008, 2010–2011, 2013–2014, 2014–2015 and 2015–2016. Severe droughts (negative SPI-6 values) occurred in 2002–2003, 2009–2010, and 2015–2016, which coincided with El Niño events (see S4 Fig). During wetter than average rainy seasons, dengue outbreaks tended to peak earlier in the dengue season, in September (see S5 Fig).

### Model framework

As dengue incidence tends to peak between September and February (see S3 Fig panel a), we defined a ‘dengue’ year as running from June to May. A hierarchical mixed model was formulated using counts of laboratory-confirmed dengue cases per month over 17 years—from June 1999 to May 2016—in Barbados as the response variable. Counts of dengue cases, y_t_ (t = 1, …, 204), were assumed to follow a negative binomial distribution,

$$y_{t}|\mu_{t}∼NegBin\left(\mu_{t}=p_{T'(t)}\rho_{t},\;\kappa \right)$$

$$log\left(\mu_{t}\right)=log\left(p_{T'(t)}\right)+log\left(\rho_{t}\right)$$ (1)

where μ_t_ is the corresponding distribution mean, which is equal to the population per 100,000 p_T'(t)_ multiplied by the unknown dengue incidence rate estimate ρ_t_ for time t. κ is the scale (or overdispersion) parameter [36]. Population effects were accounted for by including log population per 100,000 in the model as an offset at the linear predictor scale (which is assigned a coefficient of 1). The model equation can then be rearranged such that ρ_t_ is equivalent to the dengue incidence rate per 100,000 population, (y_t_ / p_T'(t)_). Then, the most suitable estimate for ρ_t_ was sought via a combination of climate covariates (included as both linear terms at individual lags and as distributed lagged nonlinear terms) and random effects to account for seasonality and unmeasured and unknown interannual variability.

First, a baseline model was formulated by including a first-order random walk latent model for month β_t'(t)_, where t'(t) = 1, …, 12 and β_1_ represents the parameter estimate for the month of June. This term helps to capture the seasonality in dengue, which is assumed to be stationary each year (monthly random effect). The first-order random walk prior allows dengue incidence rates in 1 month to depend on the previous month, to reflect both seasonality and the infectious nature of the disease. Next, exchangeable random effects for each year γ_T'(t)_ (where T'(t) = 1, …, 17 and γ_1_ represents the dengue year June 1999–May 2000) was included in the model to account for interannual variation in dengue over time (yearly random effect). This term potentially allows for changes in population immunity between outbreak years, lapses in vector control, and other slowly changing factors, such as changes in mosquito-control intensity and the introduction of new dengue virus serotypes or other viruses, which could be mistaken for dengue. Note that chikungunya and Zika viruses were introduced to Barbados in 2014 and at the end of 2015, respectively [5,37].

### Exposure–lag–response model

DLNMs [28] were used to explore and understand possible nonlinear and delayed associations between dengue incidence rates and a variety of climate impact indicators. This modelling approach is based on the definition of a cross-basis, obtained by the combination of 2 functions to flexibly model linear or nonlinear exposure–responses and the lag structure of the relationship [28]. A standard exposure–response function f(x) is defined to express the potentially nonlinear exposure–response curve along the dimension of the climate predictors. An additional lag–response function w(l) expresses instead the temporal dimension. Their combination in a bidimensional exposure–lag–response function f.w(x,l) through a cross-basis is defined to flexibly model both intensity and timing of past exposures. Meteorological variables—including monthly precipitation, minimum, mean, and maximum temperatures as well as the SPI at different time scales (1-, 3-, 6-, and 12-month averages)—were tested in the model, in turn. Note that an El Niño index was not included in the model because it is highly correlated with the SPI in Barbados (see S4 Fig), and including both would have resulted in overfitting. Each variable was included as a linear term at individual lags and as a nonlinear exposure–lag–response function. The delayed effects of the climate indicators were assessed for up to 5 months, which was determined on the basis of exploratory analysis and previous studies [27,38]. Natural cubic splines were selected for both the exposure and the lag dimensions to allow enough flexibility to capture potentially complex associations between climate indicators and their delayed impact on dengue risk.

### Model selection and estimation

Model parameters were estimated in a Bayesian framework using Integrated Nested Laplace Approximation (INLA; www.r-inla.org) [39] (see S1 Text for specification of prior and hyperprior distributions). The model was estimated in 'leave one out' cross-validation mode from June 1999 to May 2016, i.e., by excluding the month for which the prediction is valid when estimating model parameters [40]. Exploratory analysis and model selection criteria were used to select the final model, including the cross-validated (CV) log score [41] and the deviance information criterion (DIC) [42], for which smaller values indicate better fitting models. An R^2^_LR_ statistic for mixed-effects models based on a likelihood ratio test between the candidate model, and an intercept-only model was also formulated [43], where 0 ≤ R^2^_LR_ ≤ 1, with R^2^_LR_ = 1 corresponding to a perfect fit and R^2^_LR_ ≥ 0 for any reasonable model specification. R^2^_LR_ is useful as a measure of goodness-of-fit and provides an intuitive measure of the ability of the model to account for the variation in the dependent variable. The final model was selected by comparing models of increasing complexity, in terms of input variables and model structure, to the baseline model (including only the monthly random effect to account for seasonality).

### Posterior predictive distributions and out-of-sample predictions

To test the predictive ability of the model, we refitted the model 17 times, removing 1 year at a time to produce out-of-sample predictions (i.e., 17-fold cross-validation). Note that the annual random-effect term was included in the model-fitting stage to better quantify the association between climate factors and variation in dengue incidence rates. However, when producing out-of-sample predictions, no effect was estimated for the year in which the prediction was valid, hence the term does not contribute to incidence rate estimates in prediction mode. Therefore, model predictions are based solely on exposure–lag–response functions of key climate variables and the seasonality term, meaning the model can be used to predict any year not included in the model-fitting process.

To evaluate the output from the selected Bayesian hierarchical model, posterior predictive distributions of the response variable were simulated using samples from the posterior distribution of the parameters and hyperparameters in the model [44]. The posterior predictive distribution of dengue cases, y_t_, for each month (June 1999–May 2016) was estimated by drawing 1,000 random values from a negative binomial distribution with mean corresponding to the elements of μ_t_ and scale parameter corresponding to the elements of the overdispersion parameter κ, estimated from the models, estimated in 17-fold cross-validation mode. This procedure allows for uncertainty in the response variable, given the model parameters. We therefore generated 204 (17 × 12) out-of-sample posterior predictive distributions and use these to evaluate the ability of the model to produce probabilistic dengue outbreak predictions given predefined epidemic thresholds.

### Model evaluation

Dengue control programmes often monitor new cases against historical case data, which define a typical dengue transmission season [45]. While various methods for calculating epidemic thresholds are available, we chose the monthly moving thresholds of the upper quartile (75th percentile) of the distribution of observed dengue incidence. This is in line with the epidemic threshold methods described by the World Health Organization [45]. Prediction models that can detect outbreaks are crucial because they provide information to public health decision-makers who can advocate for increased disease control interventions when necessary [46]. To assess the performance of the final model against current practice, we considered the ability of both the final model and the baseline model (a submodel of the final model based only on the seasonality term) to detect outbreaks in Barbados. An outbreak threshold was calculated as the 75th percentile of the distribution of dengue cases per month between June 1999 and May 2016, excluding the ‘dengue’ year for which the prediction was valid. The probability of exceeding the moving outbreak threshold was mapped and verified with actual exceedance data. To determine an optimum decision trigger threshold for issuing outbreak alerts, we calculated a relative (or receiver) operating characteristic (ROC) curve for the binary events of exceeding the moving outbreak threshold. The ROC is a graph of the hit rate against the false alarm rate for varying decision trigger thresholds [47]. The hit rate represents the proportion of events that occurred (i.e., outbreaks) that were correctly predicted (also known as the true positive rate or sensitivity). The false alarm rate represents the proportion of events that were predicted to occur but did not occur (also known as the false positive rate or 1-specificity). We calculated the curve using probabilistic out-of-sample predictions of exceeding the moving outbreak threshold compared with the observed binary outcome. We defined a trigger alert threshold as the point on the curve closest to the point of perfect discrimination (0, 1). Performance measures, including the hit rate (probability of detection), false alarm rate, and proportion of correct predictions, were then calculated for both the final model and the baseline model.

## Results

### Model selection

As part of the model selection procedure, a range of climate variables were tested, first as linear terms for individual lags from 0 to 5 months and second as exposure–lag functions for individual climate indicators (see S3 Table, S4 Table and S5 Table). Key variables were tested with and without the interannual term to understand the relative contribution of each term to dengue incidence rate variations. The best estimate of log(ρ_t_) comprised an intercept, α, monthly, β_t'(t)_, and yearly, γ_T'(t)_, random effects (to account for seasonality and unmeasured interannual variability) and nonlinear exposure–lag functions f.w(x_it_, l) of the SPI-6, x_1t_, and Tmin, x_2t_, with lags, l, from 0 to 5 months.

$$log\left(\rho_{t}\right)=\alpha +\beta_{t'(t)}+\gamma_{T'(t)}+f.w\left(x_{1t},l\right)+f.w\left(x_{2t},l\right)$$ (2)

Table 1 shows model adequacy statistics for a series of models of increasing complexity. Firstly, a baseline model was fitted including a monthly random effect (Model 1), which accounted for 23% of the variation in dengue relative risk. Next, a yearly random effect was included (Model 2), which explained an additional 31% of the variation in addition to the annual cycle term. Exposure–lag–response functions for the selected climate factors, SPI-6, and Tmin were then added to the baseline model (individually in Model 3 and 4 and together in Model 5). This showed that 24% of the interannual variation could be explained by the key selected climate variables. The final selected model included monthly and yearly random effects and exposure–lag–response functions for both the SPI-6 and Tmin (Model 6), explaining—overall—68% of the variation in dengue incidence rates. Therefore, in addition to climate factors, yearly random effects accounted for an extra 21% of the variation in dengue incidence rates.

**Table 1:** Model adequacy results for models of increasing complexity. The CV mean logarithmic score, the DIC, and the likelihood ratio R_LR_^2^ statistic for models of increasing complexity.

Model	log(ρ_t_)	CV log score	DIC	R_LR_^2^
1	α + β_t'(t)_ Baseline (seasonal random effect)	4.46	1,801.36	0.23
2	α + β_t'(t)_ + γ_T'(t)_ Seasonal + interannual random effect	4.23	1,719.46	0.54
3	α + β_t'(t)_ + f.w(x_1t_, l) Seasonal + function of SPI-6	4.32	1,759.07	0.41
4	α + β_t'(t)_ + f.w(x_2t_, l) Seasonal + function of Tmin	4.34	1,770.52	0.34
5	α + β_t'(t)_ + f.w(x_1t_, l) + f.w(x_2t_, l) Seasonal + functions of SPI-6 + Tmin	4.28	1,742.98	0.47
6	α + β_t'(t)_ + γ_T'(t)_ + f.w(x_1t_, l) + f.w(x_2t_, l) Final (seasonal + interannual + climate functions)	4.09	1,664.94	0.68

Abbreviations: CV, cross-validated; DIC, deviance information criterion; SPI-6, 6-month Standardised Precipitation Index; Tmin, minimum temperature.

### Climate–dengue associations

**Fig 2:**
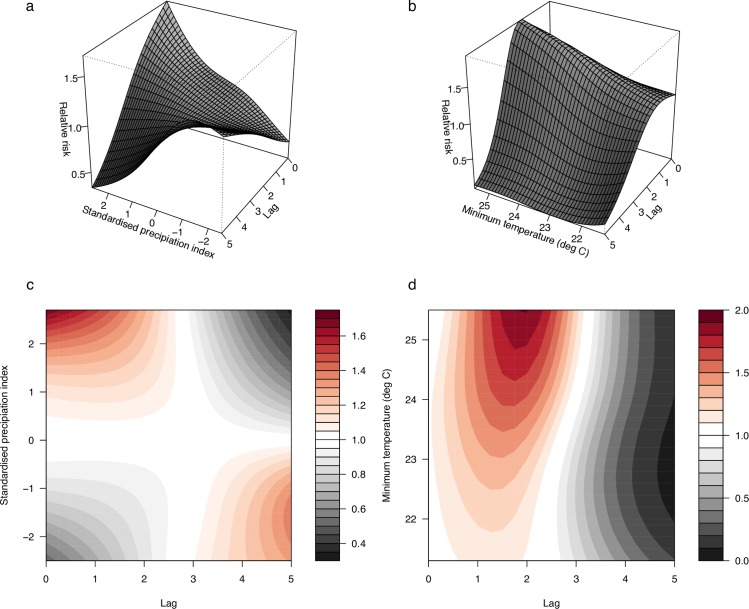
Relative risk of dengue given climatic exposures and time lags. Three-dimensional (upper panel) and contour (lower panel) plots of the exposure–lag–response association between (left) SPI-6 relative to normal conditions (SPI-6 = 0) and (right) mean temperature anomalies relative to Tmin of 20°C, at lags between 0 and 5 months. SPI-6, 6-month Standardised Precipitation Index; Tmin, minimum temperature.

**Fig 3:**
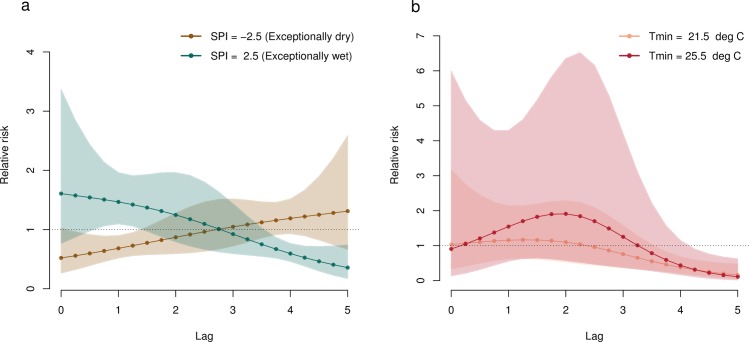
Lag–response for extreme climatic scenarios. Lag–response association for scenarios of (a) SPI-6: exceptionally dry (SPI-6 = −2.5) and exceptionally wet (SPI-6 = 2.5) conditions relative to the baseline (SPI-6 = 0) and (b) Tmin: Tmin = 21.5°C and Tmin = 25.5°C relative to the baseline (Tmin = 20°C), at lags between 0 and 5 months. SPI-6, 6-month Standardised Precipitation Index; Tmin, minimum temperature.

Associations between the selected climate variables (SPI-6 and Tmin) and dengue relative risk are presented as three-dimensional graphs and two-dimensional contour plots in Fig 2. Lag–response associations for different scenarios are shown in Fig 3. The relative risk is displayed as differences from the relative risk when SPI-6 = 0 (i.e., normal category, see S2 Table) and Tmin = 20°C (e.g., the lowest Tmin observed over the time period). The relative risk of dengue is greatest at short lead times (between 1 and 2 months) for exceptionally wet conditions (Fig 2 and Fig 3A). However, exceptionally dry conditions are associated with increased dengue relative risk 5 months later. Dengue relative risk is greatest 2 to 3 months after higher Tmin values (i.e., 25.5°C) (Fig 2 and Fig 3B). The cumulative exposure–response association across all lags for SPI-6 and Tmin show that, overall, moderately dry and warmer conditions contribute more to the relative risk (see S6 Fig).

### Detecting outbreaks

Moving outbreak thresholds were calculated as the 75th percentile of the distribution of dengue cases per month between June 1999 and May 2016, excluding the ‘dengue’ year for which the prediction was valid. We applied this moving threshold to produce out-of-sample probabilistic predictions of exceeding the outbreak threshold for all 17 years (June 1999–May 2016) as a demonstration.

**Fig 4:**
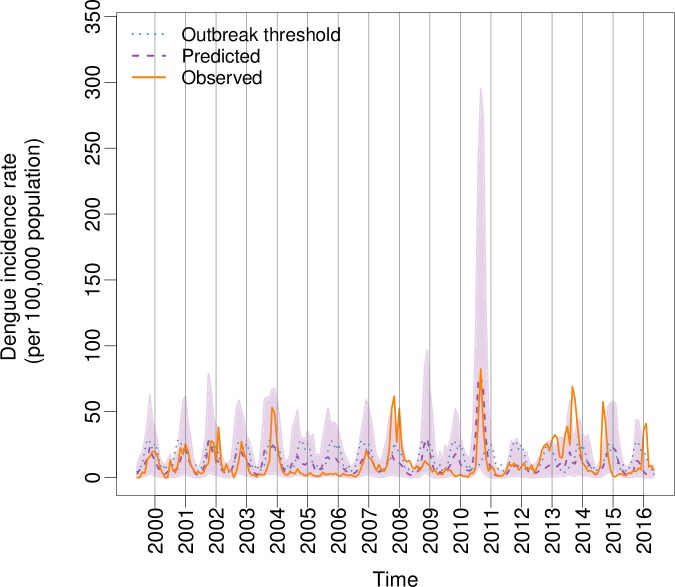
Predicted versus observed dengue incidence rates. Posterior predicted mean (dashed purple curve) and 95% prediction interval (shaded area) for dengue incidence rates (per 100,000 population) in Barbados from June 1999 to May 2016, simulated from the final model (refitted 17 times, leaving out 1 year at a time). Observed values (solid orange curve) and moving outbreak threshold (blue dotted curve) are included. Year labels are included at the start of each calendar year (e.g., in January).

**Fig 5:**
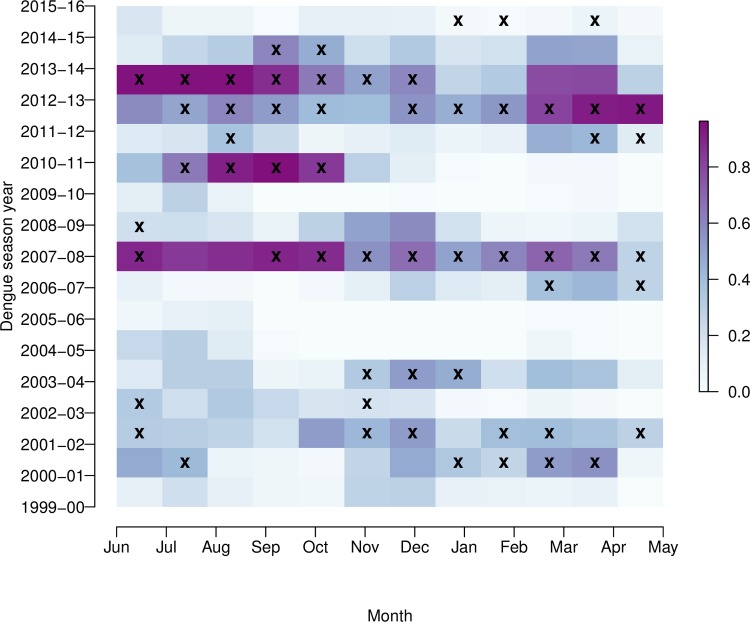
Predicted probability of exceeding moving outbreak thresholds. Probability of exceeding the moving outbreak threshold (75th percentile of observed dengue cases per month, excluding the year for which the prediction is valid) from June 1999 to May 2016, using out-of-sample predictive distributions simulated from the final model. The graduated colour bar represents the predicted probability of observing an outbreak (ranging from 0, pale colours, to 1, deep colours). Months in which the moving outbreak threshold was exceeded are marked with a cross. Note: the ‘dengue season’ year runs from June to May.

Fig 4 shows time series of observed and out-of-sample predicted dengue incidence rates (per 100,000 population) from May 1999 to June 2016. Posterior predictive mean and the upper 95% prediction (credible) interval from the final model (fitted 17 times in cross-validation mode, excluding 1 dengue year at a time), and the moving outbreak threshold (75th percentile of observed dengue incidence rates per month, excluding the year for which the prediction is valid) are also included (note, a scatter plot of observed versus posterior predictive mean dengue incidence rates from the final model is shown in S7 Fig; for comparison, posterior predictive mean dengue incidence rates from the baseline model are also shown). Fig 5 shows the probability of exceeding the moving outbreak threshold. For each time step, the proportion of the 1,000 samples simulated from the final model that exceeded the moving outbreak threshold was calculated. The deeper the colour, the greater the predicted probability of exceeding the outbreak threshold. Observed exceedance events are marked with a cross. The model was able to correctly forecast peak months and distinguish between outbreak and nonoutbreak years, except in the last 2 dengue seasons (2014–2015, 2015–2016). The model simulated a high probability (expressed as deeper colours) of exceeding the threshold in 2007–2008 and 2010–2011, and also in 2013 (Fig 5) when peaks occurred either earlier or later than expected (Fig 4). For nonoutbreak years (e.g., 2004–2005 and 2005–2006), the model correctly predicted a very low probability of outbreaks (depicted by pale colours).

The outbreak detection performance of the model is summarised in Table 2, which shows ROC and contingency table results for observed dengue cases exceeding the moving outbreak threshold. As an indicator of the quality of the forecasting system, the area under the ROC curve (AUC) was calculated as 0.90 for the final model. This indicates that the forecasting system performs significantly better than randomly guessing (AUC = 0.5). For the baseline model, AUC = 0.75 (see Table 2 and S8 Fig). The ‘optimal’ cut-off value that maximised sensitivity and specificity for the moving outbreak threshold was 30% for the final model (27% for the baseline model). Using this trigger alert threshold, we looked at a binary classification of predicted and observed outbreaks. The final model produced more hits and fewer missed events than the baseline model, with a hit rate (probability of detection) of 90% (miss rate = 10%) for the final model, compared with 79% (miss rate = 21%) for the baseline model. The final model also had a lower false alarm rate of 31% compared with 57% for the baseline model. Overall, the proportion of correctly predicted outbreaks and nonoutbreaks was 86% (see Table 2).

**Table 2:** Sensitivity and specificity results for observed dengue incidence rates exceeding the moving outbreak threshold. Summary of ROC and contingency table analysis for observed dengue incidence rates exceeding the moving outbreak threshold (75th percentile of observed dengue cases per month, excluding the year for which the prediction is valid) using out-of-sample probabilistic predictive distributions from the final and baseline models (95% CIs included in parentheses).

Performance measures	Final model	Baseline model
AUC	0.9 (0.85–0.94)	0.75 (0.71–0.79)
Probability trigger threshold	0.3	0.27
Hit rate	0.9 (0.82–0.97)	0.79 (0.69–0.9)
False alarm rate	0.31 (0.22–0.39)	0.57 (0.51–0.63)
Proportion correct	0.86 (0.81–0.91)	0.64 (0.58–0.71)

Abbreviations: AUC, area under the ROC curve; ROC, relative (receiver) operating characteristic.

## Discussion

Drought conditions were found to positively influence dengue incidence rates at longer lead times up to 5 months, while excess rainfall increased the risk at shorter lead times between 1 and 2 months. Therefore, the modelling results suggest that a drought period followed by intense rainfall 4 to 5 months later could provide optimum conditions for an imminent dengue outbreak. The use of the SPI-6, designed to monitor drought and extreme rainfall, better explained variations in dengue risk than summary statistics of measured precipitation. Tmin explained more variation in dengue incidence rates than mean or maximum temperature. Furthermore, including bidimensional exposure–lag–response functions of these indicators—rather than linear effects for individual lags—more appropriately described the climate–disease associations than traditional modelling approaches. To our knowledge, DLNM methodology has not previously been combined with a Bayesian hierarchical mixed-modelling framework to produce probabilistic predictions of exceeding dengue outbreak thresholds. To demonstrate the added value of our climate-driven dengue model, we formulated a baseline model to represent current practice (i.e., monitoring dengue cases throughout the year against historic seasonal averages). A probability trigger threshold was calculated by using the ROC curve to select an optimal cut-off value that maximised sensitivity and specificity for the moving outbreak threshold of the upper quartile of the observed dengue distribution per month. The model successfully distinguished outbreaks from nonoutbreaks, except in the last 2 dengue seasons (2014–2015, 2015–2016), with an overall proportion of correct predictions (hits and correct rejections) of 86% compared with 64% for the baseline model.

The observed effect of Tmin on dengue transmission is consistent with findings from prior studies in the Americas, which also found that Tmin was a better predictor of dengue transmission than mean or maximum temperature [48–52]. Tmin appears to be a key regulating climate parameter for dengue. In Barbados, high values of Tmin were 25.5°C, the lower end of the optimal temperature range for endemic dengue transmission (26–29°C) [13].

We hypothesise that the effect of excess rainfall and drought on dengue risk operated at different time scales due to different mechanisms associated with the availability of larval habitat and water storage in the urban environment. Following a rainfall event, the availability of larval habitat increases (e.g., rain-filled abandoned containers, rubbish), and within a relatively short period of time, eggs hatch and adult mosquito densities increase (i.e., approximately 2–3 weeks after rainfall, depending on ambient temperatures). Subsequently, the risk of arbovirus infections increases several weeks later, a lag associated with the intrinsic and extrinsic viral incubation periods.

In contrast, we observed that drought conditions, as determined by the SPI-6, were associated with dengue risk over longer periods of time (5-month lag). Barbados is among the 10 most water-scarce countries in the world [34], and water scarcity is exacerbated during periods of drought. Drought is a slow-onset climate hazard. The depletion of surface water reservoirs occurs over 6 or more months, and the depletion of groundwater reservoirs occurs at 9- to 12-month time scales. As households are alerted to water scarcity, they take measures to store water in containers around the home, increasing the availability of larval habitat for *A*. *aegypti*. As a result, we hypothesise that household water storage during drought periods may increase the risk of dengue transmission. Alternatively, we hypothesise that the increase in containers in the environment during drought may increase the risk of dengue transmission months later, when the rainy season begins and water storage is no longer a necessity. Prolonged drought could result in changes in human behaviour associated with water storage, habits that could extend beyond the period of water scarcity. In our experience, water storage containers become a greater hazard for dengue when they are not used and maintained regularly and instead serve as a ‘backup’ water source [14]. When a drought is followed by heavy rainfall, the risk of dengue could increase, as observed in 2010–2011, a year of exceptionally high dengue transmission across the Americas and an El Niño event. That year, DENV serotypes 1 and 4 were re-introduced after no circulation for several years, and an outbreak occurred with all 4 serotypes in cocirculation (2,000 suspected cases, 575 confirmed cases in Barbados) [5].

To our knowledge, there is little known about the effects of prolonged drought on dengue transmission. Some studies have shown that rainfall shortages can increase dengue risk in regions where people store water [14,15,53]. However, entomological and epidemiological field studies are needed to elucidate the mechanisms linking drought to dengue risk in Barbados. Ultimately, the effects of rainfall on dengue transmission depend on the local socioecological conditions that determine the types of larval habitat available in the environment, as well as water access and storage.

In recent decades, the Caribbean has experienced several drought events. Notable events during our study period include 2002–2003, 2009–2010, and 2015–2016. Drought and water scarcity are projected to increase in the future due to climate change [54]. To address this situation, Barbados passed building regulations that mandate the construction of rainwater storage receptacles under large new buildings. These receptacles have become ideal larval habitat for *A*. *aegypti*. Although these measures were intended to increase the capacity of the country to adapt to climate change, they may have had the unintended consequence of increasing the overall risk of arboviral diseases during any time when rainfall is collected and stored. In response, the Ministry of Health has issued recommendations to adequately cover all water storage receptacles and, if necessary, apply screen mesh material to all entry sites for small and large cisterns. Presently, the Ministry of Health is working on producing Safe Water Storage Guidelines as a reference for the public to reduce the risk of mosquito-borne diseases. This example highlights the need for intersectoral collaboration to address the complexities of climate and health in Barbados and in other SIDS.

In Barbados, over the last 5 years, there have been 3 outbreaks of dengue fever (2013–2014, 2014–2015, and 2015–2016), 1 outbreak of chikungunya virus (2014–2015), and 1 outbreak of Zika virus (2015–2016). Better management of arboviruses is a high priority for Caribbean SIDS given the burden of *A*. *aegypti*–transmitted arboviruses in the region, suitable climatic conditions for vector proliferation, and susceptible human populations, as well as the potential for transmission via widespread intra- and extraregional travel in the Caribbean [55]. To address this need, the CIMH—as part of its implementation of a regional multisectoral Early Warning Information Systems Across Climate Timescales (EWISACTs) programme—has partnered with the Caribbean Public Health Agency (CARPHA), national Ministries of Health, National Meteorological and Hydrological Services, and an international research team to support the development of regional arbovirus forecast models using climate information. This programme aims to codesign, codevelop, and codeliver sector-driven climate early warning information. One avenue for communicating warnings of dengue risk is through the Caribbean Health-Climatic Bulletin, a new joint quarterly bulletin of the CARPHA, the Pan-American Health Organization, and the CIMH [56]. The bulletin offers consensus-based expert statements of probable health outcomes extrapolated from a review of the CIMH’s suite of monitoring and forecast products contextualised to the Caribbean health context up to 3 to 6 months in advance. Integration of quantitative probabilistic forecasts of dengue risk generated by the modelling framework described in this paper represents a significant emerging research-to-operations advance that has the potential to respond to the health community’s need for empirical risk estimates for periods of operational relevance to public health. Quantitative probabilistic forecasts of disease risk will enhance the quality of warning information and provide improved guidance for decision-making.

Climate anomalies in the Caribbean are associated with tropical Atlantic and Pacific sea surface temperatures, which are usually well predictable at seasonal time scales [57]. This gives useful predictive skill to regional and seasonal climate forecast models, especially seasonal temperature and SPI-based outlooks with up to 6 months lead time. Previous studies have shown that seasonal climate forecasts combined with dengue risk prediction models can extend predictive lead time from a few months to nearly a year [36,58], thus indicating the potential to develop public health interventions based on climate information in the Caribbean.

**Fig 6:**
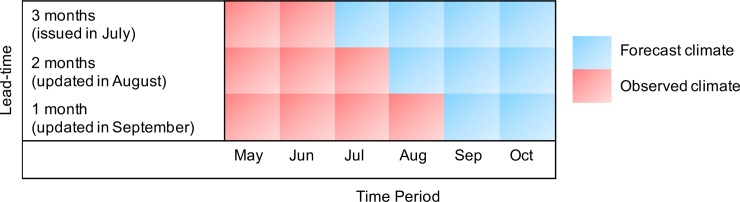
The type of climate information required for a dengue forecast in a given target month. Schematic to show the type (e.g., observed or forecast) of climate information (e.g., SPI-6 and Tmin variables, lags 0 to 5) used to produce a dengue forecast for the target month of October. The first forecast would be issued 3 months ahead of the target (i.e., 3-month lead time), in July, using observed climate data (for SPI-6 and Tmin) for May and June and forecast climate data for July, August, September, and October. The forecast would then be updated in August (i.e., 2-month lead time) using observed climate for July and updated climate forecasts for August, September, and October. In September, 1 month before the target (i.e., 1-month lead time), the dengue forecast would be updated again, using observed climate from May to August and updated climate forecasts for September and October. SPI-6, 6-month Standardised Precipitation Index; Tmin, minimum temperature.

In practice, the CIMH could run this dengue model using their seasonal climate forecast products. Results could be updated on a monthly basis as the target month is approached. This should reduce forecast uncertainty as one more month of climate observations becomes available and one fewer month of climate forecasts is needed. CariCOF’s SPI-6-based drought outlooks have a prediction lead time of 3 months. For example, CariCOF’s SPI-6-based drought outlook issued in July, which estimates the drought situation by the end of October, could be used to predict the dengue risk in October (target month). Observed SPI-6 data for May (i.e., calculated from the observed rainfall totals from December of the previous year to May of the current year) and June (i.e., 4 and 5 months before the target month, to capture the long-lag impacts on dengue risk) would be used along with forecast SPI-6 conditions from July to October (i.e., lags of 0–3 months with respect to the target month). This would produce a 3-month lead dengue forecast (see Fig 6). In August, the dengue forecast for October could be updated—using the new observed SPI-6 value for July and updated SPI-6 forecasts for August (i.e., an SPI-6 calculated from the sum of observed rainfall totals from March to July and forecast rainfall totals for August) to October—to produce a 2-month-ahead dengue forecast for October (Fig 6), and so on. The dengue model parameter estimates could be updated on an annual basis as new dengue incidence and climate data become available.

Seasonal forecast models can contribute to reducing the risk of disease outbreaks by increasing preparation time to better allocate scarce resources. To date, there are few localised studies in this region that quantify the associations between subseasonal to seasonal forecasts of climate and dengue risk outcomes. In the absence of a robust evidence base, the operational use of climate information for predicting increased dengue risk remains an untapped opportunity to support climate-driven dengue early warning information systems. This is an area of further investigation, along with the transferability of this framework to model and predict the risk of dengue and other arboviruses in other Caribbean countries.

There are several limitations of the study. In Barbados, the absolute number of dengue cases across the island is relatively small, even in outbreak years (i.e., 1,140 confirmed cases in 2013, 488 confirmed cases in 2014, and 587 confirmed cases in 2016). The time series is relatively long compared with previously studied dengue records in other countries. However, it is still limited for statistical modelling purposes because our knowledge of outbreak dynamics is based on the behaviour of only a few outbreaks. Ideally, data on vector control activities and other interventions or policy changes would be included in the model. In the absence of such data, monthly and yearly random effects are included in the model to try and ensure that the variance in the predictions includes uncertainty that could be generated by these unknown features of the disease system. However, while yearly effects are useful to identify missing information and better quantify the contribution of explanatory variables to the relative risk, they cannot contribute to out-of-sample predictions unless prior assumptions about the year ahead can be made.

The model failed to predict the peak and magnitude of the last 2 dengue transmission seasons, which coincided with the first epidemics of chikungunya and Zika. The emergence of these viruses, which are transmitted by the same mosquito vector, likely altered diagnostic, reporting, and healthcare-seeking practices, and we suspect that this might have affected the model performance for the last 2 dengue seasons in 2015 and 2016. This may also be true in other countries and territories across Latin America and the Caribbean during this time period. Dengue virus, chikungunya virus, and Zika virus have a similar clinical presentation (e.g., mild febrile illness, rash, joint pain), and differential diagnosis is challenging without molecular diagnostics (e.g., PCR) due to cross-reactivity of the serological assays. Only after September 2016 was Barbados able to test suspected arbovirus infections using a triplex PCR; prior to then, a subset of samples was sent for confirmation to CARPHA’s diagnostic laboratory in Trinidad and Tobago. The emergence of Zika virus may have also changed healthcare-seeking behaviour, with more women seeking diagnostics for mild febrile illness due to concerns about Zika congenital syndrome [37], resulting in an increase in the detection of dengue and Zika infections in women. The cocirculation of dengue, chikungunya, and Zika in the region poses a challenge for forecasting future risk, unless high-quality seroprevalence data can be incorporated in the model [36].

Overall, the final model had a lower false alarm rate and a lower miss rate than the baseline model. However, the model did result in false alarms 31% of the time. Repeatedly issuing false alarms can result in a lack of trust from the general public. However, in practice, false alarms (i.e., issuing a high probability alert of a dengue outbreak) can, in fact, result from effective interventions in response to the alert. Furthermore, when dealing with detrimental health events, such as disease outbreaks, false alarms are preferable to missed events.

The objective of this study was to select a model to infer delayed and nonlinear impacts of climate on dengue risk and predict the probability of exceeding an outbreak threshold relevant to the Ministry of Health in Barbados and other islands in the Caribbean. Forecasts of the probability of exceeding outbreak thresholds allow decision-makers to quantify the level of certainty of the model predictions. However, when using predefined outbreak and alarm trigger thresholds, the translation of probabilities into discrete warnings might not always reflect the predictive power of the model [46]. Outbreak and alarm trigger thresholds need to be carefully chosen to reflect the capacity of the public health system to respond to imminent outbreaks.

## Conclusion

We are working towards implementing a climate-based early warning system for arboviral diseases in all Caribbean islands and territories. Not only does this study contribute to enriching the knowledge base, it represents a considerable opportunity to translate investment in health–climate research into practice to improve national and regional health outcomes. The goal is to enhance the health warnings issued in the quarterly Caribbean Health-Climatic Bulletin with quantitative probabilistic forecasts of disease risk rather than expert statements on probable health outcomes. Integration of an early warning information product into national and sectoral planning and practice has the potential to reverse the upward trend of new infections of arboviral diseases, which currently undermines the productivity and sustainable development of SIDS in the Caribbean.

## Supporting information

S1 Fig Site map and location of meteorological stations in Barbados. Map of Caribbean (left) and Barbados showing population (middle) and elevation (right) and the locations of the 2 main meteorological stations (CIMH and GAIA). This figure was created in ArcGIS version 10.3.1 [59] using shapefiles from the GADM database of Global Administrative Areas version 2.8, freely available at gadm.org [60]. CIMH, Caribbean Institute for Meteorology and Hydrology; GAIA, Grantley Adams International Airport.
(TIF)

S2 Fig Smoothed rainfall seasonality at the CIMH synoptic station in Barbados. The chance of a wet day (i.e., a calendar day with >0.85 mm; left panel) and the average rainfall intensity on a wet day for each Julian day of the year (right panel). Source: modified from Trotman and colleagues [20]. CIMH, Caribbean Institute for Meteorology and Hydrology.
(TIF)

S3 Fig Annual cycle of dengue incidence rates and meteorological station data. Annual cycle of (a) dengue incidence rate (per 100,000 population), (b) precipitation (mm/month), and (c) Tmin (°C) averaged over CIMH and GAIA weather stations. CIMH, Caribbean Institute for Meteorology and Hydrology; GAIA, Grantley Adams International Airport; Tmin, minimum temperature.
(TIF)

S4 Fig Annual cycle of the SPI-6 and the Oceanic Niño Index. Annual cycle of (a) SPI-6 and (b) Oceanic Niño Index, defined as the 3-month running-mean sea surface temperature departures from average in the Niño 3.4 region (120–170° W, 5° S-5° N), from June 1999 to May 2016. SPI-6, 6-month Standardised Precipitation Index.
(TIF)

S5 Fig Annual cycle of dengue cases in Barbados during drier than normal, near-normal, and wetter than normal seasons. Annual cycle of dengue cases given tercile categories of the SPI-6: drier than normal (solid curve), normal (dashed curve), and wetter than normal (dotted curve). During wetter than average years, dengue cases tended to peak earlier in the season, in September, whereas dry years coincided with late-season peaks. SPI-6, 6-month Standardised Precipitation Index.
(TIF)

S6 Fig Overall cumulative exposure–response. Exposure–response association across all lags (0 to 5 months) for (a) the SPI-6 relative to the baseline SPI-6 = 0 and (b) Tmin relative to the baseline Tmin = 20°C. SPI-6, 6-month standardised precipitation index; Tmin, minimum temperature.
(TIF)

S7 Fig Observed versus predicted dengue incidence rates. Observed versus posterior predicted mean dengue incidence rates (per 100,000 population) from the final model (purple circles) and the baseline model (red squares). Note: logarithmic scale.
(TIF)

S8 Fig ROC curves to define alarm trigger threshold. ROC curve for binary event of dengue incidence rates exceeding the moving outbreak threshold (75th percentile of observed dengue incidence rates per month, excluding the year for which the prediction is valid) for (a) final model (without year effect contribution) and (b) baseline model. Numbers indicate values of probability thresholds along the curve, and the purple circle indicates the position of an ‘optimal’ ROC cut-off (alarm trigger threshold), defined as the point on the curve closest to the point of perfect discrimination (0, 1). Note: false alarms are a desired outcome when the predicted probability of threshold exceedance is very low. ROC, relative (receiver) operating characteristic.
(TIF)

S1 Table SPI values and precipitation intensities [35]. SPI, Standardised Precipitation Index.
(DOCX)

S2 Table SPI classification used in the Caribbean from January 2011. SPI, Standardised Precipitation Index.
(DOCX)

S3 Table Model adequacy results for single-lag univariate models. Summary statistics for a model including monthly and yearly random effects and (a) SPI-6 and (b) Tmin at individual lags of 0 to 5 months: posterior mean, 95% CIs, the CV mean logarithmic score, the DIC, and the likelihood ratio R_LR_^2^ statistic. CI, credible interval; CV, cross-validated; DIC, deviance information criterion; SPI-6, 6-month Standardised Precipitation Index; Tmin, minimum temperature.
(DOCX)

S4 Table SPI variable at different time scales. The CV mean logarithmic score, the DIC, and the likelihood ratio R_LR_^2^ statistic for models including monthly and yearly random effects and an exposure–lag–response function with time lags from 0 to 5 months for the SPI averaged over 1 month, 3 months, 6 months, and 12 months). The best indicator is shaded in grey. CV, cross-validated; DIC, deviance information criterion; SPI, Standardised Precipitation Index.
(DOCX)

S5 Table Climate variable selection. The CV mean logarithmic score, the DIC, and the likelihood ratio R_LR_^2^ statistic for models including monthly and yearly random effects and an exposure–lag–response function with time lags from 0 to 5 months for individual climate variables: precipitation, SPI (1-month, 3-month, 6-month, and 12-month), Tmin, mean temperature, and maximum temperature. CV, cross-validated; DIC, deviance information criterion; SPI, Standardised Precipitation Index; Tmin, minimum temperature.
(DOCX)

S1 Text Prior and hyperprior distribution specification. (DOCX)

## Abbreviations

AUC: area under the ROC curve
CariCOF: Caribbean Climate Outlook Forum
CARPHA: Caribbean Public Health Agency
CDPMN: Caribbean Drought and Precipitation Monitoring Network
CIMH: Caribbean Institute for Meteorology and Hydrology
CV: cross-validated
DENV: dengue virus
DIC: deviance information criterion
DLNM: distributed lag nonlinear model
ENSO: El Niño Southern Oscillation
EWISACTs: Early Warning Information Systems Across Climate Timescales
GAIA: Grantley Adams International Airport
INLA: Integrated Nested Laplace Approximation
NS1: nonstructural protein 1
RCC: Regional Climate Centre
ROC: relative (receiver) operating characteristic
SIDS: Small Island Developing States
SPI: Standardised Precipitation Index
SPI-6: SPI averaged over a 6-month period
Tmin: minimum temperature
