# Supplementary figures and images for: Nonlinear and delayed impacts of climate on dengue risk in Barbados: A modelling study
Source: PLoS Med. 2018 Jul 17;15(7):e1002613. doi: 10.1371/journal.pmed.1002613 (PMC6049902; doi:10.1371/journal.pmed.1002613)

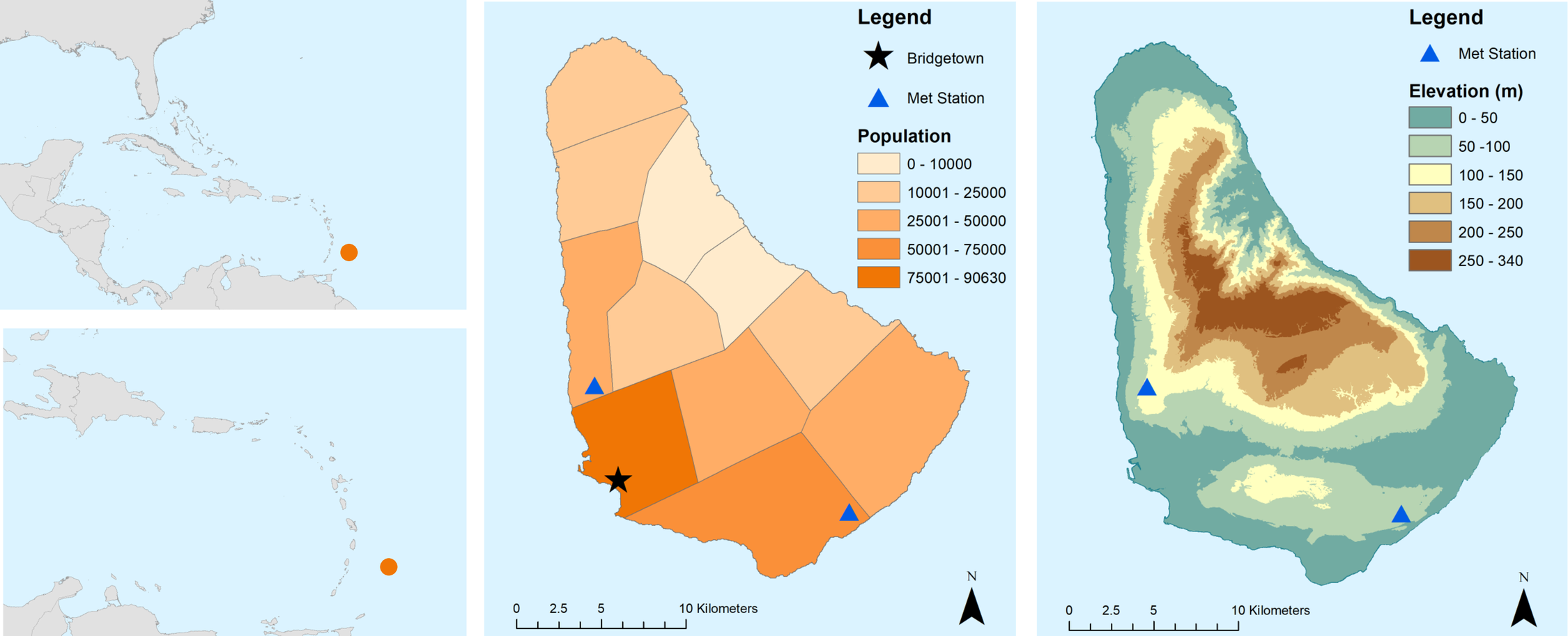

Supplement: S1 Fig — Map of Caribbean (left) and Barbados showing population (middle) and elevation (right) and the locations of the 2 main meteorological stations (CIMH and GAIA). This figure was created in ArcGIS version 10.3.1 [59] using shapefiles from the GADM database of Global Administrative Areas version 2.8, freely available at gadm.org [60]. CIMH, Caribbean Institute for Meteorology and Hydrology; GAIA, Grantley Adams International Airport. (TIF) [file pmed.1002613.s001.tif]

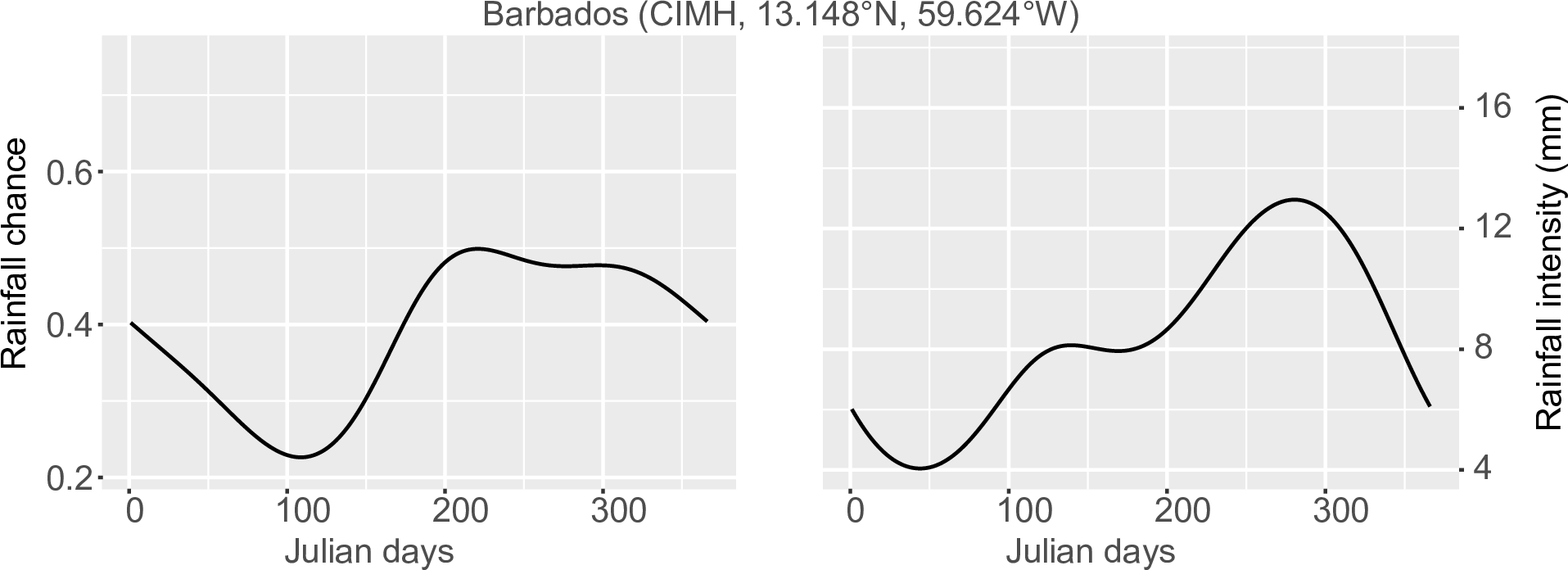

Supplement: S2 Fig — The chance of a wet day (i.e., a calendar day with >0.85 mm; left panel) and the average rainfall intensity on a wet day for each Julian day of the year (right panel). Source: modified from Trotman and colleagues [20]. CIMH, Caribbean Institute for Meteorology and Hydrology. (TIF) [file pmed.1002613.s002.tif]

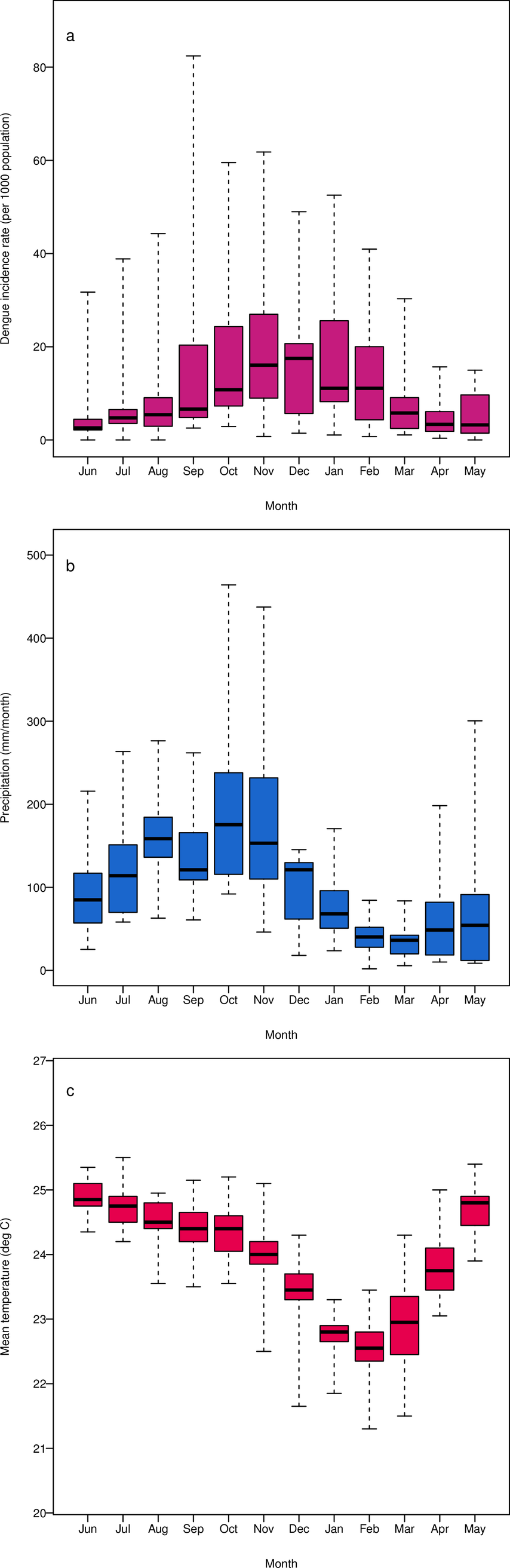

Supplement: S3 Fig — Annual cycle of (a) dengue incidence rate (per 100,000 population), (b) precipitation (mm/month), and (c) Tmin (°C) averaged over CIMH and GAIA weather stations. CIMH, Caribbean Institute for Meteorology and Hydrology; GAIA, Grantley Adams International Airport; Tmin, minimum temperature. (TIF) [file pmed.1002613.s003.tif]

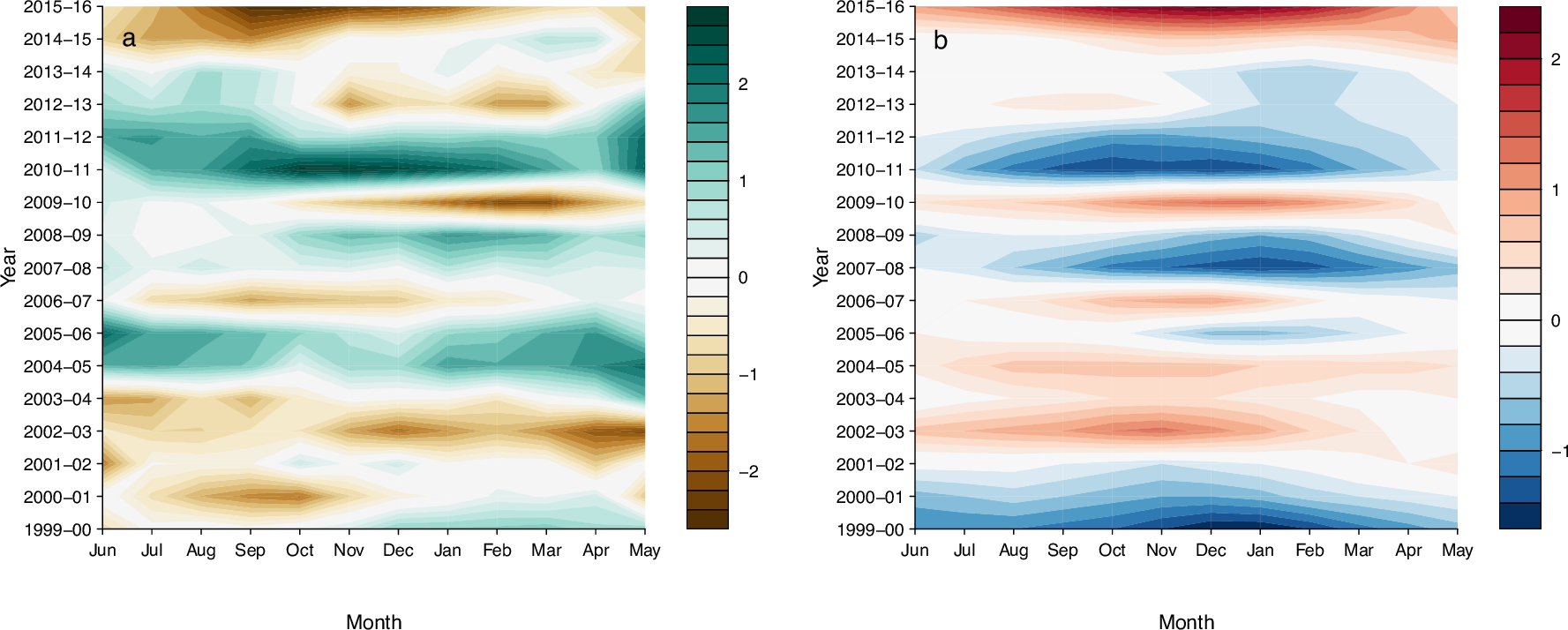

Supplement: S4 Fig — Annual cycle of (a) SPI-6 and (b) Oceanic Niño Index, defined as the 3-month running-mean sea surface temperature departures from average in the Niño 3.4 region (120–170° W, 5° S-5° N), from June 1999 to May 2016. SPI-6, 6-month Standardised Precipitation Index. (TIF) [file pmed.1002613.s004.tif]

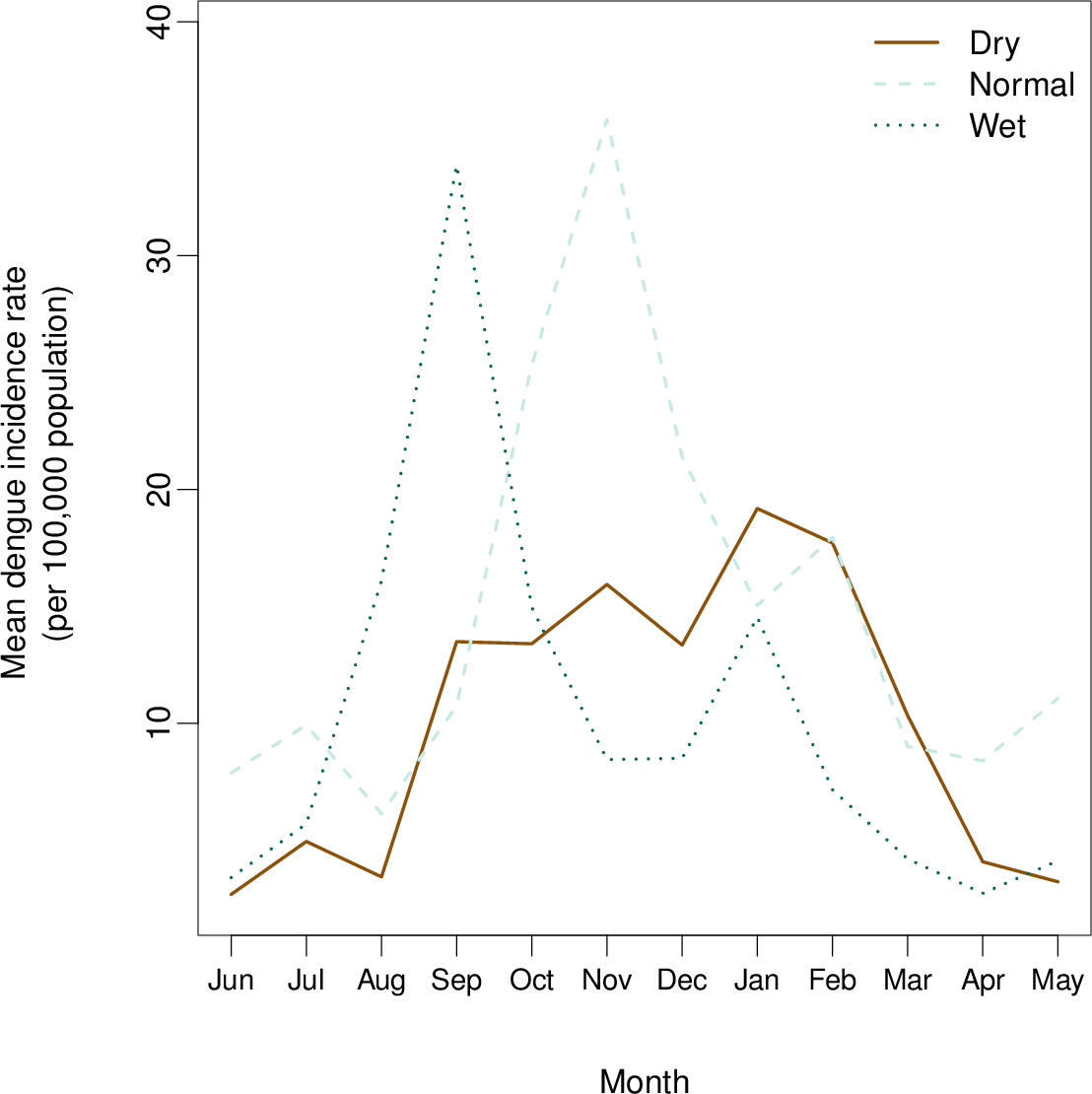

Supplement: S5 Fig — Annual cycle of dengue cases given tercile categories of the SPI-6: drier than normal (solid curve), normal (dashed curve), and wetter than normal (dotted curve). During wetter than average years, dengue cases tended to peak earlier in the season, in September, whereas dry years coincided with late-season peaks. SPI-6, 6-month Standardised Precipitation Index. (TIF) [file pmed.1002613.s005.tif]

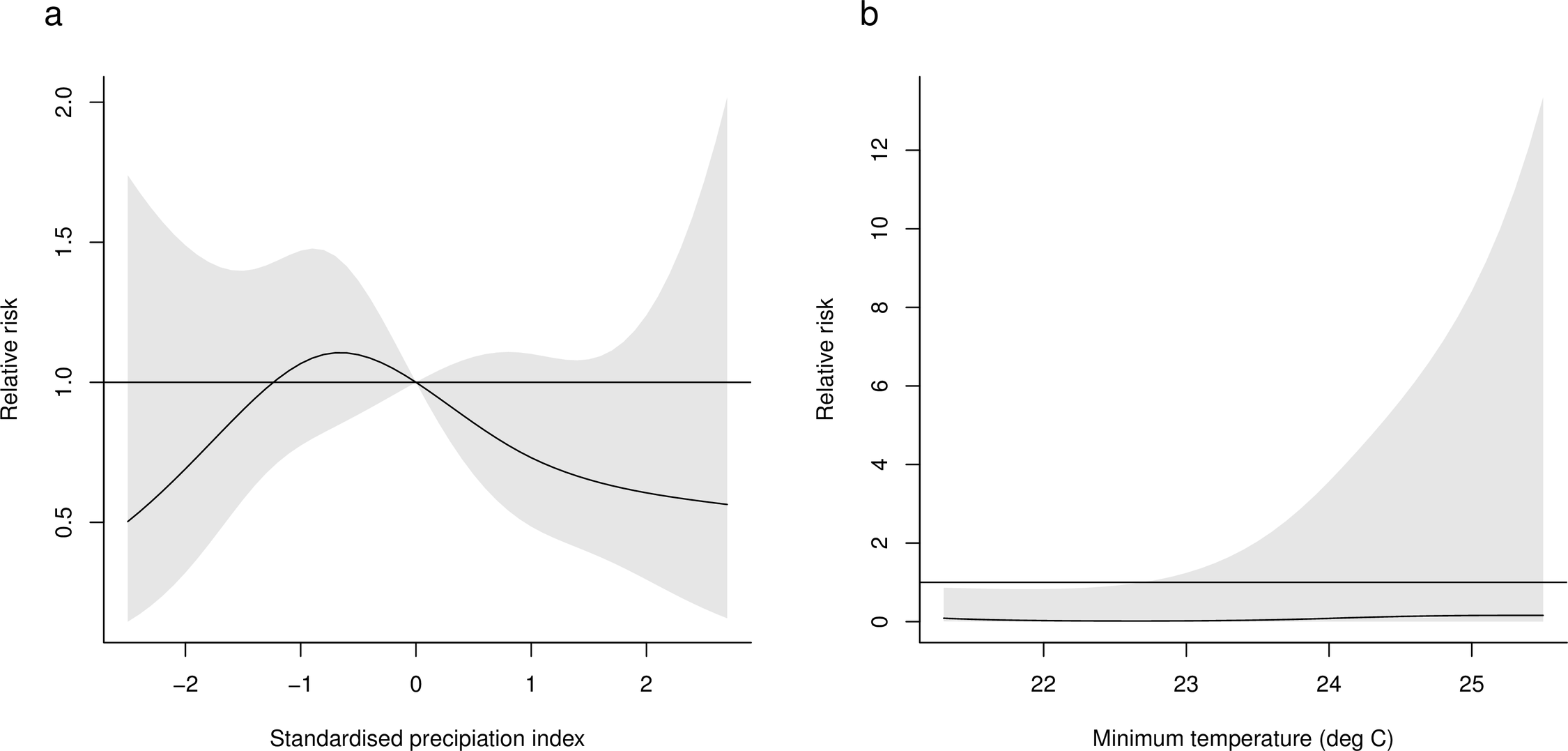

Supplement: S6 Fig — Exposure–response association across all lags (0 to 5 months) for (a) the SPI-6 relative to the baseline SPI-6 = 0 and (b) Tmin relative to the baseline Tmin = 20°C. SPI-6, 6-month standardised precipitation index; Tmin, minimum temperature. (TIF) [file pmed.1002613.s006.tif]

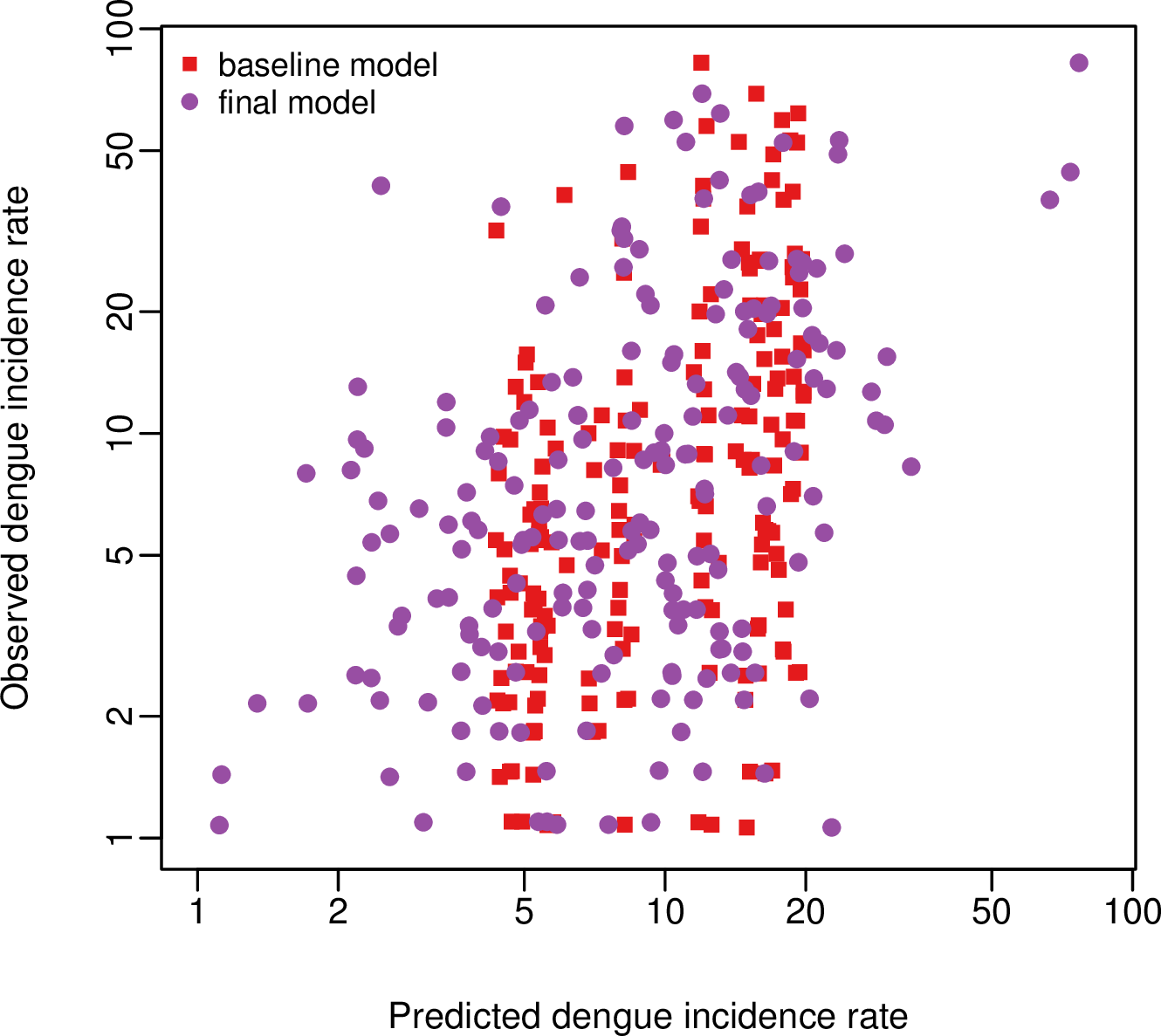

Supplement: S7 Fig — Observed versus posterior predicted mean dengue incidence rates (per 100,000 population) from the final model (purple circles) and the baseline model (red squares). Note: logarithmic scale. (TIF) [file pmed.1002613.s007.tif]

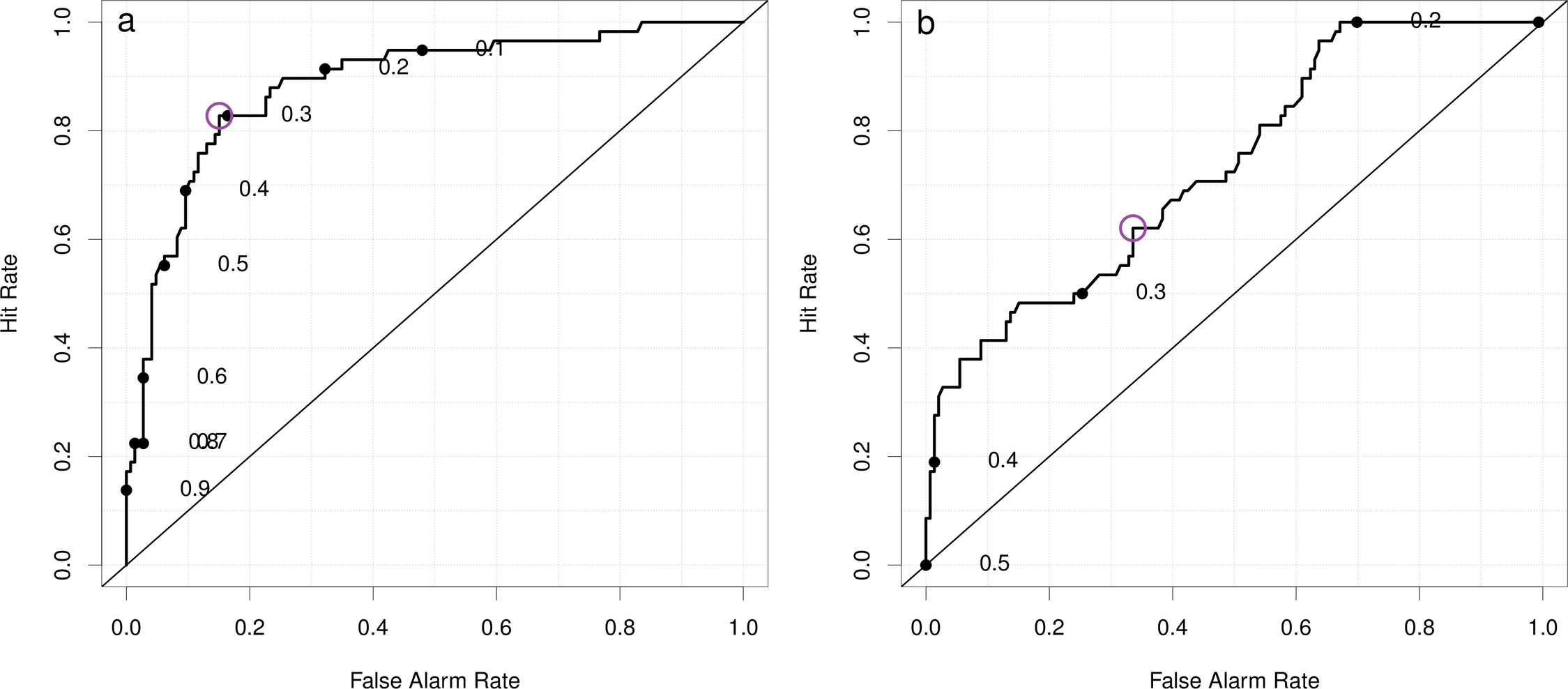

Supplement: S8 Fig — ROC curve for binary event of dengue incidence rates exceeding the moving outbreak threshold (75th percentile of observed dengue incidence rates per month, excluding the year for which the prediction is valid) for (a) final model (without year effect contribution) and (b) baseline model. Numbers indicate values of probability thresholds along the curve, and the purple circle indicates the position of an ‘optimal’ ROC cut-off (alarm trigger threshold), defined as the point on the curve closest to the point of perfect discrimination (0, 1). Note: false alarms are a desired outcome when the predicted probability of threshold exceedance is very low. ROC, relative (receiver) operating characteristic. (TIF) [file pmed.1002613.s008.tif]
