# Supplementary material for: Nonlinear and delayed impacts of climate on dengue risk in Barbados: A modelling study
Source: PLoS Med. 2018 Jul 17;15(7):e1002613. doi: 10.1371/journal.pmed.1002613 (PMC6049902; doi:10.1371/journal.pmed.1002613)
Supplement: S1 Table — SPI, Standardised Precipitation Index. (DOCX) [file pmed.1002613.s009.docx]

**S1 Table.** SPI values and precipitation intensities [35]. SPI, Standardised Precipitation Index.

| **SPI** | **Category** | **Probability (%)** |
| --- | --- | --- |
| 2.0 + | Extremely wet | 2.3 |
| 1.5 to 1.99 | Very wet | 4.4 |
| 1.0 to 1.49 | Moderately wet | 9.2 |
| -0.99 to 0.99 | Near normal | 68.2 |
| -1.0 to -1.49 | Moderately dry | 9.2 |
| -1.5 to -1.99 | Severely dry | 4.4 |
| -2.0 and less | Extremely dry | 2.3 |
