# Supplementary material for: Nonlinear and delayed impacts of climate on dengue risk in Barbados: A modelling study
Source: PLoS Med. 2018 Jul 17;15(7):e1002613. doi: 10.1371/journal.pmed.1002613 (PMC6049902; doi:10.1371/journal.pmed.1002613)
Supplement: S2 Table — SPI, Standardised Precipitation Index. (DOCX) [file pmed.1002613.s010.docx]

**S2 Table.** SPI classification used in the Caribbean from January 2011. SPI, Standardised Precipitation Index.

| **SPI value** | **Category** | **SPI value** | **Impact** |
| --- | --- | --- | --- |
| -0.50 to -0.01 | Normal | 0.50 to 0.01 | Normal |
| -0.80 to -0.51 | Abnormally dry | 0.80 to 0.51 | Abnormally wet |
| -1.30 to -0.81 | Moderately dry | 1.30 to 0.81 | Moderately wet |
| -1.60 to -1.31 | Severely dry | 1.60 to 1.31 | Very wet |
| -2.00 to -1.61 | Extremely dry | 2.00 to 1.61 | Extremely wet |
| ≤ -2.01 | Exceptionally dry | ≥ 2.01 | Exceptionally wet |
