# Supplementary material for: Nonlinear and delayed impacts of climate on dengue risk in Barbados: A modelling study
Source: PLoS Med. 2018 Jul 17;15(7):e1002613. doi: 10.1371/journal.pmed.1002613 (PMC6049902; doi:10.1371/journal.pmed.1002613)
Supplement: S3 Table — Summary statistics for a model including monthly and yearly random effects and (a) SPI-6 and (b) Tmin at individual lags of 0 to 5 months: posterior mean, 95% CIs, the CV mean logarithmic score, the DIC, and the likelihood ratio RLR2 statistic. CI, credible interval; CV, cross-validated; DIC, deviance information criterion; SPI-6, 6-month Standardised Precipitation Index; Tmin, minimum temperature. (DOCX) [file pmed.1002613.s011.docx]

**S3 Table. Model adequacy results for single-lag univariate models.**

Summary statistics for a model including monthly and yearly random effects and (a) SPI-6 and (b) Tmin at individual lags of 0 to 5 months: posterior mean, 95% CIs, the CV mean logarithmic score, the DIC, and the likelihood ratio R_LR_^2^ statistic. CI, credible interval; CV, cross-validated; DIC, deviance information criterion; SPI-6, 6-month Standardised Precipitation Index; Tmin, minimum temperature.

(a)

| **Lag** | **Mean** | **Lower 95% CI** | **upper 95% CI** | **CV log score** | **DIC** | **R_LR_^2^** |
| --- | --- | --- | --- | --- | --- | --- |
| Lag 0 | 0.172 | 0 | 0.347 | 4.218 | 1714.076 | 0.557 |
| Lag 1 | 0.031 | -0.131 | 0.195 | 4.231 | 1720.492 | 0.541 |
| Lag 2 | -0.118 | -0.269 | 0.032 | 4.228 | 1719.576 | 0.542 |
| Lag 3 | -0.286 | -0.421 | -0.152 | 4.188 | 1704.736 | 0.575 |
| Lag 4 | -0.358 | -0.478 | -0.239 | 4.148 | 1688.156 | 0.61 |
| Lag 5 | -0.393 | -0.509 | -0.276 | 4.13 | 1680.537 | 0.625 |

(b)

| **Lag** | **Mean** | **Lower 95% CI** | **upper 95% CI** | **CV log score** | **DIC** | **R_LR_^2^** |
| --- | --- | --- | --- | --- | --- | --- |
| Lag 0 | -0.226 | -0.458 | 0.008 | 4.224 | 1718.367 | 0.543 |
| Lag 1 | -0.169 | -0.437 | 0.093 | 4.226 | 1718.519 | 0.545 |
| Lag 2 | 0.316 | 0.058 | 0.563 | 4.224 | 1718.917 | 0.54 |
| Lag 3 | 0.548 | 0.413 | 0.681 | 4.23 | 1723.898 | 0.5 |
| Lag 4 | 0.554 | 0.424 | 0.684 | 4.231 | 1721.738 | 0.503 |
| Lag 5 | 0.395 | 0.166 | 0.605 | 4.228 | 1720.083 | 0.532 |
