# Supplementary material for: Nonlinear and delayed impacts of climate on dengue risk in Barbados: A modelling study
Source: PLoS Med. 2018 Jul 17;15(7):e1002613. doi: 10.1371/journal.pmed.1002613 (PMC6049902; doi:10.1371/journal.pmed.1002613)
Supplement: S4 Table — The CV mean logarithmic score, the DIC, and the likelihood ratio RLR2 statistic for models including monthly and yearly random effects and an exposure–lag–response function with time lags from 0 to 5 months for the SPI averaged over 1 month, 3 months, 6 months, and 12 months). The best indicator is shaded in grey. CV, cross-validated; DIC, deviance information criterion; SPI, Standardised Precipitation Index. (DOCX) [file pmed.1002613.s012.docx]

**S4 Table. SPI variable at different time scales.**

The CV mean logarithmic score, the DIC, and the likelihood ratio R_LR_^2^ statistic for models including monthly and yearly random effects and an exposure–lag–response function with time lags from 0 to 5 months for the SPI averaged over 1 month, 3 months, 6 months, and 12 months). The best indicator is shaded in grey. CV, cross-validated; DIC, deviance information criterion; SPI, Standardised Precipitation Index.

| **Climate variables** | **CV log score** | **DIC** | **R_LR_^2^** |
| --- | --- | --- | --- |
| Standardised precipitation index (1-month) | 4.22 | 1713.56 | 0.59 |
| Standardised precipitation index (3-month) | 4.22 | 1713.57 | 0.59 |
| Standardised precipitation index (6-month) | 4.13 | 1678.67 | 0.65 |
| Standardised precipitation index (12-month) | 4.16 | 1693.48 | 0.63 |
