# Supplementary material for: Nonlinear and delayed impacts of climate on dengue risk in Barbados: A modelling study
Source: PLoS Med. 2018 Jul 17;15(7):e1002613. doi: 10.1371/journal.pmed.1002613 (PMC6049902; doi:10.1371/journal.pmed.1002613)
Supplement: S5 Table — The CV mean logarithmic score, the DIC, and the likelihood ratio RLR2 statistic for models including monthly and yearly random effects and an exposure–lag–response function with time lags from 0 to 5 months for individual climate variables: precipitation, SPI (1-month, 3-month, 6-month, and 12-month), Tmin, mean temperature, and maximum temperature. CV, cross-validated; DIC, deviance information criterion; SPI, Standardised Precipitation Index; Tmin, minimum temperature. (DOCX) [file pmed.1002613.s013.docx]

**S5 Table. Climate variable selection.**

The CV mean logarithmic score, the DIC, and the likelihood ratio R_LR_^2^ statistic for models including monthly and yearly random effects and an exposure–lag–response function with time lags from 0 to 5 months for individual climate variables: precipitation, SPI (1-month, 3-month, 6-month, and 12-month), Tmin, mean temperature, and maximum temperature. CV, cross-validated; DIC, deviance information criterion; SPI, Standardised Precipitation Index; Tmin, minimum temperature.

| **Climate variables** | **CV log score** | **DIC** | **R_LR_^2^** |
| --- | --- | --- | --- |
| Precipitation | 4.16 | 1690.22 | 0.61 |
| Standardised precipitation index (6-month) | 4.13 | 1678.67 | 0.65 |
| Minimum temperature | 4.19 | 1705.79 | 0.58 |
| Mean temperature | 4.2 | 1709.06 | 0.57 |
| Maximum temperature | 4.22 | 1715.31 | 0.55 |
