# Supplementary material for: Nonlinear and delayed impacts of climate on dengue risk in Barbados: A modelling study
Source: PLoS Med. 2018 Jul 17;15(7):e1002613. doi: 10.1371/journal.pmed.1002613 (PMC6049902; doi:10.1371/journal.pmed.1002613)
Supplement: S1 Text — (DOCX) [file pmed.1002613.s014.docx]

**Text S1. Prior and hyperprior distribution specification**

Model parameters were estimated in a Bayesian framework, using Integrated Nested Laplace Approximation (INLA, [www.r-inla.org](http://www.r-inla.org)) in R version 3.4.2. Parameter uncertainty is accounted for by assigning prior distributions to the parameters. Autocorrelated random effects for each month of the dengue year (from June to May) were included to account for the annual cycle of dengue. The month effect β_t'(t)_ was assigned a random walk or first difference prior distribution, in which each effect is derived from the immediately preceding effect, β_t'(t)_ - β_t'(t)−1_ ∼ N(0, σ^2^_β_), t’(t) = 1, . . . , 12 where β_1_ represents the parameter estimate for the month of June. For model fitting purposes, independent and exchangeable random effects γ_T'(t)_ , T’(t)=1,…,17 were included for each ‘dengue’ year (from June to May) using a Gaussian distribution with zero mean and large variance for the unstructured prior γ_T'(t)_ ∼ N (0, σ^2^γ) (Note, for out-of-sample predictions the year indicator was set to null for the year we were trying to predict). For both random effect terms, we assigned the R-INLA default hyperparameter to the precision (τ = 1/σ^2^), that is τ ~Gamma(a, b) with shape parameter a = 1 and inverse-scale parameter b =1⋅10^-5^_._
